# Birth mode is associated with development of atopic dermatitis in infancy and early childhood

**DOI:** 10.1016/j.jacig.2023.100104

**Published:** 2023-03-29

**Authors:** Sveinung T. Hoel, Johanna Wiik, Karin C.L. Carlsen, Kim M.A. Endre, Hrefna Katrín Gudmundsdóttir, Guttorm Haugen, Angela Hoyer, Christine Monceyron Jonassen, Marissa LeBlanc, Björn Nordlund, Knut Rudi, Håvard O. Skjerven, Anne Cathrine Staff, Gunilla Hedlin, Cilla Söderhäll, Riyas Vettukattil, Hilde Aaneland, Eva M. Rehbinder

**Affiliations:** a Institute of Clinical Medicine, Faculty of Medicine, University of Oslo, Oslo, Norway; b Department of Gynecology and Obstetrics, Østfold Hospital Trust, Kalnes, Norway; c Department of Obstetrics and Gynecology, Institute of Clinical Sciences, Sahlgrenska Academy, University of Gothenburg, Gothenburg, Sweden; d Department of Obstetrics and Gynecology, Sahlgrenska University Hospital, Region Västra Götaland, Gothenburg, Sweden; e Division of Pediatric and Adolescent Medicine, Oslo University Hospital, Oslo, Norway; f Department of Dermatology and Vaenerology, Oslo University Hospital, Oslo, Norway; g Division of Obstetrics and Gynaecology, Oslo University Hospital, Oslo, Norway; h Oslo Centre for Biostatistics and Epidemiology, Oslo University Hospital, Oslo, Norway; i Astrid Lindgren Children’s Hospital, Karolinska University Hospital, Stockholm, Sweden; j Department of Women’s and Children’s Health, Karolinska Institute, Stockholm, Sweden; k Centre for Laboratory Medicine, Østfold Hospital Trust, Grålum, Norway; l Department of Chemistry, Biotechnology and Food Science, Norwegian University of Life Sciences, Ås, Norway; m Faculty of Chemistry, Biotechnology and Food Science, Norwegian University of Life Sciences, Ås, Norway

**Keywords:** Atopic dermatitis, early onset atopic dermatitis, persistent atopic dermatitis, birth mode, caesarean section, C-section, vaginal birth, water birth, obstetrics

## Abstract

**Background:**

Birth by caesarean section (CS) is associated with development of allergic diseases, but its role in the development of atopic dermatitis (AD) is less convincing.

**Objective:**

Our primary aim was to determine if birth mode was associated with AD in 3-year-olds and secondarily to determine if birth mode was associated with early onset and/or persistent AD in the first 3 years of life.

**Methods:**

We included 2129 mother–child pairs from the Scandinavian population-based prospective PreventADALL cohort with information on birth mode including vaginal birth, either traditional (81.3%) or in water (4.0%), and CS before (6.3%) and after (8.5%) onset of labor. We defined early onset AD as eczema at 3 months and AD diagnosis by 3 years of age. Persistent AD was defined as eczema both in the first year and at 3 years of age, together with an AD diagnosis by 3 years of age.

**Results:**

AD was diagnosed at 3, 6, 12, 24, and/or 36 months in 531 children (25%). Compared to vaginal delivery, CS was overall associated with increased odds of AD by 3 years of age, with adjusted odds ratio (95% confidence interval) of 1.33 (1.02-1.74), and higher odds of early onset AD (1.63, 1.06-2.48). The highest odds for early onset AD were observed in infants born by CS after onset of labor (1.83, 1.09-3.07). Birth mode was not associated with persistent AD.

**Conclusion:**

CS was associated with increased odds of AD by 3 years of age, particularly in infants presenting with eczema at 3 months of age.

Atopic dermatitis (AD), also known as atopic eczema, is a common and chronic relapsing-remitting disease that occurs in 15% to 20% of children and 7% of adults.^1^^,^^2^ The disease leads to red, itchy skin, which can be severely troublesome for those affected. The strongest known inherited predicting factor for AD are loss-of-function mutations in the gene coding for filaggrin, *FLG.* The filaggrin protein connects keratin fibers in epithelial cells and strengthens the skin barrier.^3^ Mutations in *FLG* increase the risk for AD,^4^, ^5^, ^6^, ^7^, ^8^, ^9^ especially for early onset AD.^4^

While caesarean sections (CSs) can save lives and prevent perinatal mortality and morbidity,^10^ they are accompanied by both short- and long-term risks.^10^^,^^11^ CS is associated with allergic diseases,^12^, ^13^, ^14^ but results on the association between birth mode and development and risk of AD show no evidence of association. Four prospective cohort studies and 1 retrospective register-based study found no evidence that CS increased the risk of AD in children aged between 3 months and 6 years.^13^^,^^15^, ^16^, ^17^, ^18^

Delivery by CS has commonly been compared to vaginal delivery in studies of allergic disease of the offspring, without differentiation between acute and elective CS or between intact or ruptured fetal membranes,^10^, ^11^, ^12^, ^13^, ^14^^,^^16^^,^^17^^,^^19^^,^^20^ and thus not accounting for the infant’s degree of exposure to the maternal vaginal microbiota. Birth in water can potentially affect the initial microbial colonization of the newborn,^21^ although the effect on allergic diseases and AD has yet to be investigated.

It is not clear if birth mode may influence the risk of early versus later onset of AD in childhood.^15^ However, a previous study from the Preventing Atopic Dermatitis and Allergies in Children (PreventADALL) cohort revealed that elective CS was associated with increased risk of eczema at 3 months of age,^22^ supporting the hypothesis that birth mode might alter the development of AD, possibly increasing the risk of early onset AD.

Therefore, we wanted to explore the effect of CS before onset of labor (before start of contractions and membrane rupture) and CS after onset of labor (after start of contractions and/or membrane rupture) on AD. Our primary aim was to determine if birth mode was associated with AD diagnosis in the first 3 years of life, and secondarily was to determine if birth mode was associated with early onset or persistent AD. Thirdly, we aimed to investigate whether the results would change when we stratified children according to *FLG* loss-of-function mutations.

## Methods

### Study design

The PreventADALL study is both a factorial, multicenter, cluster-randomized, controlled trial and an observational, population-based mother–child birth cohort study in Norway (Oslo University Hospital and Østfold Hospital Trust, Kalnes) and Sweden (Karolinska University Hospital, Stockholm) (see the Online Repository at www.jaci-global.org). Participants were recruited antenatally at the 18-week routine ultrasound examination. The women completed electronic questionnaires around weeks 18 and 34, reporting sociodemographic and lifestyle factors as well as health and family history. The study team registered birth mode along with general obstetric information from electronic hospital records after delivery in formalized study protocols. In addition, further obstetric details were obtained through individual medical chart reviews in Norway and through the Swedish Pregnancy Register in Sweden.^23^ The children attended investigations with clinical examinations at 3, 6, 12, 24, and 36 months of age, including evaluation of their skin by trained medical personnel.^24^

Informed consent was signed by the mothers at enrollment, and by both parents at inclusion of the infant. The PreventADALL study was approved by the Regional Committee for Medical and Health Research Ethics in South-Eastern Norway (2014/518) and in Sweden (2014/2242-31/4) and was registered at ClinicalTrials.gov (NCT02449850).

### Study population

**Fig 1:**
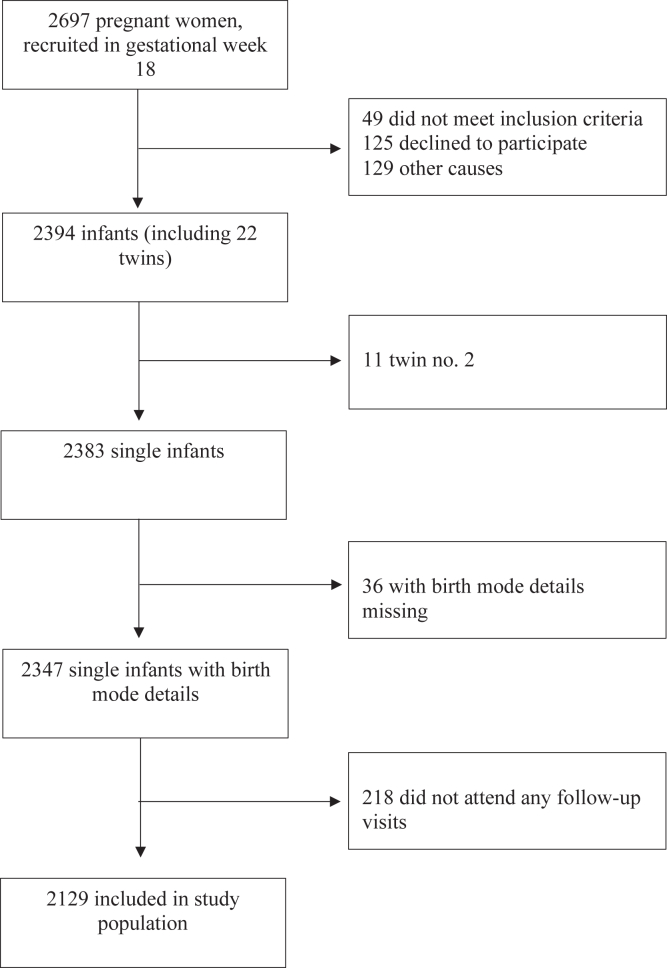
Flowchart showing selection process of study population in current substudy from PreventADALL cohort.

From December 2014 to October 2016, a total of 2697 pregnant women were enrolled onto the PreventADALL study.^24^ All women planning to give birth in Oslo and Østfold as well as women from several maternity clinics in Stockholm region were invited to participate in the study when attending the 18-week gestational age (GA) routine ultrasound examination. Their infants born at a GA of at least 35 weeks without any serious illnesses were enrolled during the first or second day of life. Exclusion criteria were pregnancy with more than 2 fetuses, lack of sufficient Scandinavian language skill, plans to move outside reasonable travel distance within 1 year after delivery, and presence of severe maternal, fetal, or neonatal disease. Fetuses with severe malformations or disease and infants born before 35 weeks’ gestation were excluded. Our study population includes 2129 infant participants attending at least one of the 3-, 6-, 12-, 24-, and 36-month investigations with available information on birth mode. The second twins of 11 twin pairs were excluded in the current study (Fig 1).

### Exposure

Birth mode was divided into 2 or 4 categories, as follows. First, the 2 categories of birth mode were (1) any vaginal birth, including traditional nonassisted, assisted (instrumental), and vaginal water birth, and (2) any CS, including both CS before onset of labor (before start of contractions and membrane rupture) and CS after onset of labor (after start of contractions and/or membrane rupture). Second, the 4 categories of birth mode were based on fetal exposure to vaginal microbiota and included (1) traditional vaginal birth (both nonassisted and assisted), (2) vaginal water birth, (3) CS before onset of labor (before start of regular contractions and/or membrane rupture), and (4) CS after onset of labor (after start of regular contractions and/or membrane rupture).

For *FLG* analyses, DNA was isolated from blood (umbilical cord blood sampled at birth or by venous puncture at 12 months) and genotyped using TaqMan-based allelic discrimination assay (Applied Biosystems; Thermo Fisher Scientific, Waltham, Mass), as previously described.^25^ If genotyping analysis showed “undetermined” results, we labeled the values as missing. We defined infants with *FLG* mutations (“mutation yes”) as being carriers of any of R501X, 2282del4, and R2447X mutations of the *FLG* gene—the most common loss-of-function mutations in the European population.

### Outcome

The primary outcome was AD by 3 years of age, and secondary outcomes were early onset AD and persistent AD. AD by 3 years of age was defined as being diagnosed with AD at any time by age 3 years. Early onset AD was defined as the presence of eczema at 3 months’ investigation and being diagnosed with AD at any time by age 3 years. Persistent AD was defined as the presence of eczema at any time during the first year of life (at 3, 6, or 12 months’ investigations) as well as at 3 years’ investigation, and additionally being diagnosed with AD at any time by age 3 years.

AD was diagnosed by fulfilling the UK Working Party’s diagnostic criteria^26^ at the investigations at 3, 6, 12, 24, and 36 months and/or the Hanifin and Rajka diagnostic criteria^27^ at 12, 24, and 36 months by a trained medical doctor.

### Statistical analysis

Odds ratios, 95% confidence intervals (CIs), and *P* values are presented as numbers with 2 decimals, except significant numbers, which are presented as full ciphers. A significance level of .05 was applied throughout. Chi-square test for categorical variables and independent-sample *t* test for numeric variables were conducted to discover any significant differences in baseline characteristics between the selected study population and the rest of the PreventADALL cohort.

Unadjusted and adjusted binary and multinomial logistic regression models were used to assess the association between birth mode and AD. Either the 2 birth-mode model including any vaginal births or any CS or the 4 birth-mode model including traditional vaginal birth, vaginal water birth, and CS before and after onset of labor was used. The studied outcomes were (1) AD versus no AD by 3 years of age, (2) early onset AD versus no early onset AD or no AD, and (3) persistent AD versus nonpersistent AD or no AD. Adjustments were made for GA (as a continuous variable), parity (nulliparous or not), and infant’s sex, which were identified as relevant confounders by drawing a direct acyclic graph (see Fig E1 in the Online Repository at www.jaci-global.org).

Missing data were handled through best-case imputations, assuming that active eczema would be motivational for attending follow-ups. No attendance at a given follow-up was therefore defined as “no AD” and “no eczema.” We chose best-case imputations for regression analyses, while full case analyses were included as sensitivity analyses. A complete description of the missing case handling is provided in the Online Repository at www.jaci-global.org.

Subanalyses to investigate the effect of infant *FLG* mutations and birth mode on AD were performed. To evaluate the interacting effect of *FLG* mutation status on the association between CS and AD, an interaction analysis was used, both adjusted and unadjusted (see Table E1 in the Online Repository at www.jaci-global.org), as well as a logistic regression model comparing vaginal birth and CS with or without *FLG* mutations (Table I).

**Table I:** Logistic regression model for *FLG* mutations and birth mode for outcomes adjusted for sex, GA, and parity

Characteristic	Significance	aOR	95% CI
AD by 36 months			
Vaginal, no *FLG* mutation (reference)	0		
CS, no *FLG* mutation	.016	1.41	1.066-1.868
Vaginal with *FLG* mutation	<.001	2.82	1.967-4.038
CS with *FLG* mutation	.09	2.32	0.89-6.05
Early onset analysis: Compared to no AD			
AD with no early onset∗			
Vaginal, no *FLG* mutation (reference)	0		
CS, no *FLG* mutation	.15	1.27	0.92-1.76
Vaginal with *FLG* mutation	<.001	2.264	1.487-3.450
CS with *FLG* mutation	.328	1.78	0.56-5.65
Early onset AD†			
Vaginal, no *FLG* mutation (reference)	0		
CS, no *FLG* mutation	.009	1.812	1.160-2.840
Vaginal with *FLG* mutation	<.001	4.358	2.650-7.180
CS with *FLG* mutation	.041	3.89	1.060-14.26
Persistent AD analysis: Compared to no AD			
Nonpersistent AD‡			
Vaginal, no *FLG* mutation (reference)	0		
CS, no *FLG* mutation	.13	1.29	0.93-1.78
Vaginal with *FLG* mutation	<.001	2.412	1.59-3.65
CS with *FLG* mutation	.018	3.21	1.23-8.40
Persistent AD§			
Vaginal, no *FLG* mutation (reference)	0		
CS, no *FLG* mutation	.02	1.734	1.11-2.72
Vaginal with *FLG* mutation	<.001	3.90	2.34-6.51
CS with *FLG* mutation	NA	NA	NA

*NA,* Not applicable.

∗ No eczema at 3 months but AD by 36 months.

† Eczema at 3 months and AD by 36 months.

‡ No observed eczema by age 12 months or at 36 months’ investigation, and AD diagnosed by 36 months.

§ Eczema both by age 12 months and at 36 months’ investigation, and AD diagnosis by 36 months.

To explore the interacting effect of AD heredity on the association between CS and AD, interaction analyses were performed, both adjusted and unadjusted (see Table E2 in the Online Repository at www.jaci-global.org). AD heredity was present if one or both parents had an AD diagnosis. Subanalyses with adjusted logistic regression stratified for AD heredity (see Table E3 in the Online Repository) were performed in addition to subanalyses comparing vaginal birth and CS for the AD outcomes with adjusted logistic regression, also including AD heredity in the adjustments (see Table E4 in the Online Repository).

Statistical analyses were conducted by SPSS Statistics v27 software (IBM, Armonk, NY).

## Results

Among the 2129 included infants, 52.9% were boys, mean (SD) birth weight was 3574 (474) g, and mean (SD) GA at birth was 40 (1.3) weeks. Table II shows baseline characteristics of the study population comparing infants with and without AD by 36 months of age. AD was diagnosed at 3, 6, 12, 24, and/or 36 months in 531 children (25%).

**Table II:** Baseline characteristics of study population comparing infants with and without AD by 36 months of age

Characteristic	AD in infant by 36 months			*P* value∗
	No	Yes	Total	
No. of subjects	1598	531	2129	
Sex (n = 2129)				.488
Male	839 (52.5)	288 (54.2)	1127 (52.9)	
Female	759 (47.5)	243 (45.8)	1002 (47.1)	
Mother origin (n = 1937)				.030
Norway	945 (64.9)	344 (71.5)	1289 (66.5)	
Sweden	357 (24.5)	86 (17.9)	443 (22.9)	
Nordic	20 (1.4)	5 (1.0)	25 (1.3)	
Other Europe	53 (3.6)	22 (4.6)	75 (3.9)	
Rest of world	81 (5.6)	24 (5.0)	105 (5.4)	
Father origin (n = 1896)				.056
Norway	916 (64.4)	331 (70.0)	1247 (65.8)	
Sweden	348 (24.5)	87 (18.4)	435 (22.9)	
Nordic	20 (1.4)	6 (1.3)	26 (1.4)	
Rest of world	139 (9.8)	49 (10.4)	188 (9.9)	
Mother diagnosed with AD (n = 1940)†				<.001
Yes	255 (17.5)	131 (27.2)	386 (19.9)	
No	1204 (82.5)	350 (72.8)	1554 (80.1)	
Father diagnosed with AD (n = 1946)‡				<.001
Yes	134 (9.2)	70 (14.3)	204 (10.5)	
No	1282 (87.9)	388 (79.5)	1670 (85.8)	
Unknown	42 (2.9)	30 (6.1)	72 (3.7)	
Birth mode (n = 2129)				.249
Vaginal regular	1315 (82.3)	416 (78.3)	1731 (81.3)	
Vaginal in water	61 (3.8)	24 (4.5)	85 (4.0)	
CS before onset of labor	94 (5.9)	39 (7.3)	133 (6.2)	
CS after onset of labor	128 (8.0)	52 (9.8)	180 (8.5)	
Birth mode (n = 2129)				.067
Vaginal birth	1376 (86.1)	440 (82.9)	1816 (85.3)	
CS	222 (13.9)	91 (17.1)	313 (14.7)	
Age of mother (years)† (n = 2129)	32.3 [4.1]	32.5 [3.7]	32.4 [4.1]	.199
Age of father (years)† (n = 1812)	34.4 [5.4]	34.8 [5.5]	34.7 [5.4]	.623
Maternal BMI (kg/m^2^) before pregnancy (n = 2075)	22.9 [3.6]	23.3 [3.6]	23.0 [3.6]	.290
GA at birth (weeks) (n = 2125)	40 [1.4]	40 [1.3]	40 [1.3]	.130
Birth weight (g) (n = 2123)	3565 [490]	3591 [455]	3574 [474]	.168

Data are presented as nos. (%) or means [SDs] unless otherwise indicated. *Nordic* refers to origins from Nordic regions other than Norway and Sweden (ie, Finland, Iceland, or Denmark). *BMI,* Body mass index.

∗ Chi-square test significance.

† From enrollment questionnaire.

‡ From 36-week questionnaire.

Overall, 313 children (14.7%) were born by CS, while 1731 (81.3%) were born by traditional vaginal delivery, 85 (4.0%) by vaginal delivery in water, 133 (6.3%) by CS before onset of labor, and 180 (8.5%) by CS after onset of labor (Table II).

**Fig 2:**
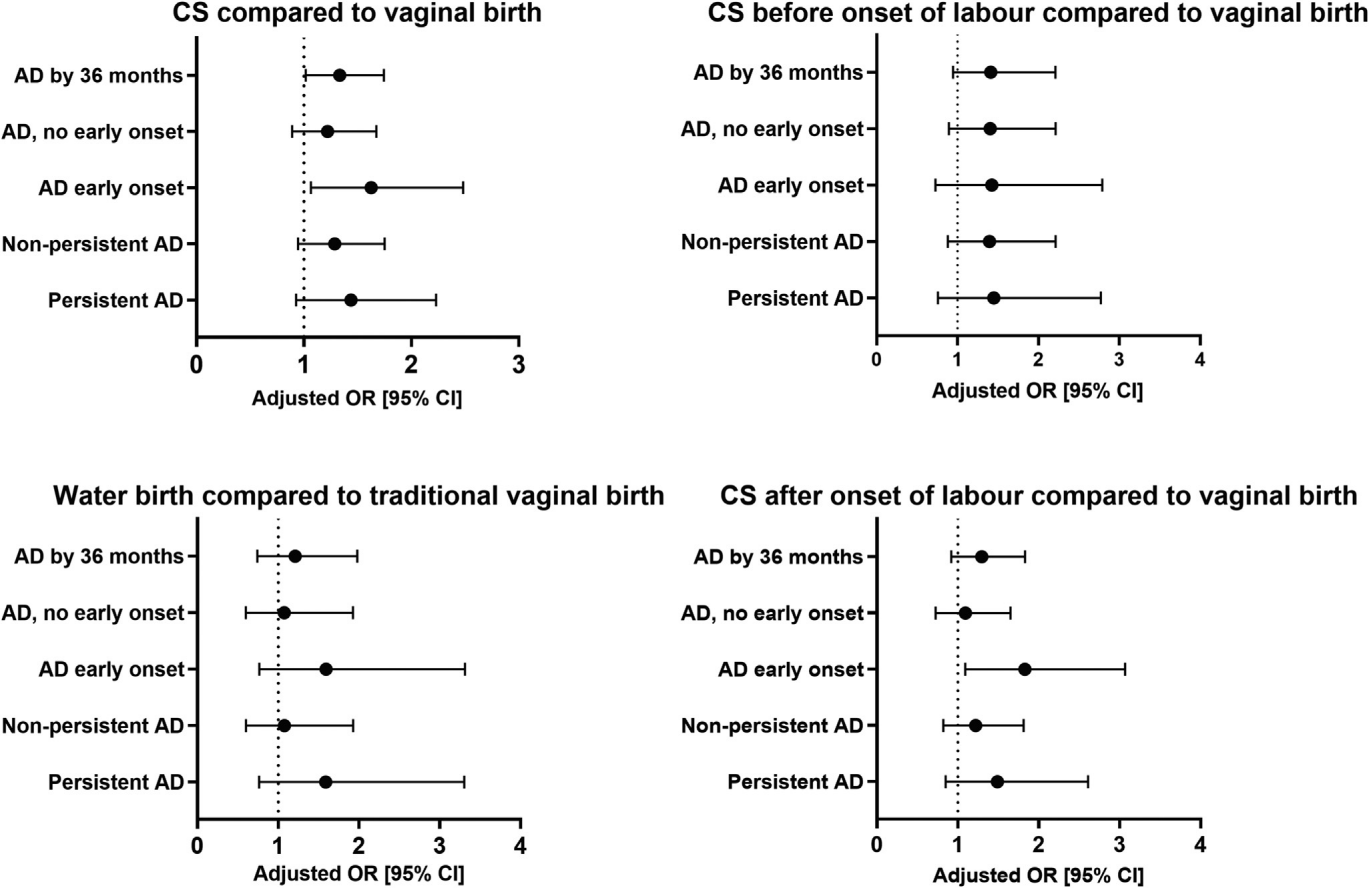
Forest plot for CS overall, water birth, CS before onset of labor, and CS after onset of labor compared to traditional vaginal birth, adjusted for sex, GA, and parity. CS before onset of labor was defined as CS before start of contractions and membrane rupture; CS after onset of labor was defined as CS after start of contractions and/or membrane rupture.

Infants born by CS had significantly increased odds of being diagnosed with AD by 3 years of age (adjusted odds ratio [aOR] 1.33, 95% CI 1.02-1.74, *P* = .037) compared to infants born by vaginal birth. In the model with 4 different birth categories, there was no significant association between any birth mode and AD by 3 years (Table III, Fig 2).

**Table III:** Outcomes of binary and multinomial logistic regression model for 2 and 4 birth-mode models, adjusted for sex, GA, and parity

Birth-mode model∗	Significance	aOR	95% CI
CS compared to vaginal birth			
AD by 36 months	.037	1.33	1.02-1.74
AD with no early onset	.22	1.22	0.89-1.67
Early onset AD	.025	1.63	1.064-2.48
Nonpersistent AD	.31	1.20	0.85-1.69
Persistent AD	.11	1.44	0.93-2.23
Vaginal water birth compared to regular vaginal birth			
AD by 36 months	.44	1.21	0.74-1.98
AD with no early onset	.81	1.07	0.60-1.93
Early onset AD	.21	1.59	0.77-3.31
Nonpersistent AD	.81	1.08	0.6-1.93
Persistent AD	.22	1.59	0.76-3.30
CS before onset of labor compared to vaginal birth			
AD by 36 months	.09	1.41	0.95-2.11
AD with no early onset	.14	1.40	0.89-2.21
Early onset AD	.3	1.42	0.73-2.79
Nonpersistent AD	.16	1.4	0.88-2.21
Persistent AD	.26	1.45	0.76-2.77
CS after onset of labor compared to vaginal birth			
AD by 36 months	.14	1.30	0.92-1.83
AD with no early onset	.67	1.09	0.72-1.65
Early onset AD	.020	1.830	1.090-3.070
Nonpersistent AD	.33	1.2	0.82-1.81
Persistent AD	.17	1.49	0.85-2.61

∗ AD with no early onset indicates no eczema at 3 months, but AD by 36 months; early onset AD, eczema at 3 months and AD by 36 months; nonpersistent AD, no observed eczema by 12 months of age or at 36 months’ investigation, and AD diagnosed by 36 months; and persistent AD, eczema both by 12 months of age and at 36 months’ investigation, and AD diagnosis by 36 months.

Infants born by CS had a significantly increased odds of early onset AD (aOR 1.63, 95% CI 1.06-2.48, *P* = .025) compared to infants born vaginally. In the analysis of the 4 different birth modes, only CS after onset of labor was significantly associated with early onset AD (aOR 1.83, 95% CI 1.09-3.07, *P* = .020) compared to traditional vaginal birth (Table III).

**Table IV:** Cross-tabulation of 2 birth-mode models

Characteristic†	Vaginal birth	CS	*P* value∗
No. of subjects	1816	313	
AD by 36 months			.067
No	1376 (75.8)	222 (70.9)	
Yes	440 (24.2)	91 (29.1)	
AD by time of onset			.095
No AD	1376 (75.8)	222 (70.9)	
AD with no early onset	315 (17.3)	60 (19.2)	
Early onset AD	125 (6.9)	31 (9.9)	
AD by persistence			.167
No AD	1376 (75.8)	222 (70.9)	
Nonpersistent AD	315 (17.3)	63 (20.1)	
Persistent AD	125 (6.9)	28 (8.9)	

Data are presented as nos. (%) unless otherwise indicated.

∗ Chi-square test significance.

† AD with no early onset indicates no eczema at 3 months, but AD by 36 months; early onset AD, eczema at 3 months and AD by 36 months; nonpersistent AD, no observed eczema by 12 months of age or at 36 months’ investigation, and AD diagnosed by 36 months; and persistent AD, eczema both by 12 months of age and at 36 months’ investigation, and AD diagnosis by 36 months.

Birth mode was not significantly associated with persistent AD in any model (Tables III, IV, and V).

**Table V:** Cross-tabulations of 4 birth-mode models

Characteristic†	Vaginal regular	Vaginal in water	CS before onset of labor	CS after onset of labor	*P* value∗
No. of subjects	1731	85	133	180	
AD by 36 months					.249
No	1315 (76.0)	61 (71.8)	94 (70.7)	128 (71.1)	
Yes	416 (24.0)	24 (28.2)	39 (29.3)	52 (28.9)	
AD by time of onset					.255
No AD	1315 (76.0)	61 (71.8)	94 (70.7)	128 (71.1)	
AD with no early onset	300 (17.3)	15 (17.6)	28 (21.1)	32 (17.8)	
Early onset AD	116 (6.7)	9 (10.6)	11 (8.3)	20 (11.1)	
AD by persistence					.483
No AD	1315 (76.0)	61 (71.8)	94 (70.7)	128 (71.1)	
Nonpersistent AD	300 (17.3)	15 (17.6)	27 (20.3)	36 (20.0)	
Persistent AD	116 (6.7)	9 (10.6)	12 (9.0)	16 (8.9)	

Data are presented as nos. (%) unless otherwise indicated.

∗ Chi-square test significance.

† AD with no early onset indicates no eczema at 3 months, but AD by 36 months; early onset AD, eczema at 3 months and AD by 36 months; nonpersistent AD, no observed eczema by 12 months of age or at 36 months’ investigation, and AD diagnosed by 36 months; and persistent AD, eczema both by 12 months of age and at 36 months’ investigation, and AD diagnosis by 36 months.

**Fig 3:**
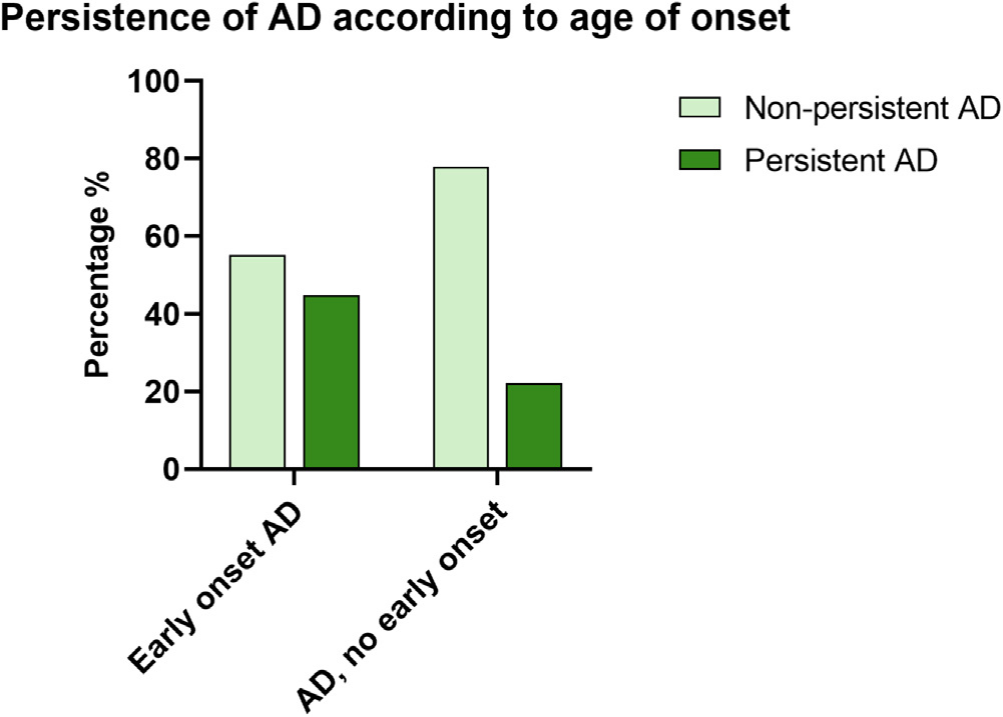
Grouped column graph showing persistence of AD according to age at onset. AD was defined as follows: AD with no early onset indicates no eczema at 3 months but AD by 36 months; early onset AD, eczema at 3 months and AD by 36 months; nonpersistent AD, no observed eczema by 12 months of age or at 36 months’ investigation, and AD diagnosed by 36 months; and persistent AD, eczema by both 12 months and at 36 months, and AD diagnosis by 36 months.

Children with *FLG* mutations born vaginally had significantly increased odds for AD regardless of onset and persistence of AD compared to vaginally delivered children without *FLG* mutation (Fig 3). The small group of CS-born children carrying an *FLG* mutation (n = 18) showed increased odds for early onset AD and nonpersistent AD compared to vaginally born children without *FLG* mutations but was not significantly associated with increased odds of AD by 3 years of age (Table I).

Interaction analyses on birth mode and AD heredity and *FLG* showed that the effect of CS on AD depended on neither AD heredity overall (Table E2) nor *FLG* mutation (Table E1).

## Discussion

In a general population of more than 2000 children, we found that CS increased the overall odds of being diagnosed with AD by 3 years of age, with a higher risk for early onset AD related to CS performed after onset of labor. The odds of AD by 3 years of age were increased by CS, regardless of heredity for AD and *FLG* mutation status. We did not find any association between CS and persistent AD.

Our results conflict a population-based prospective study of 459 children in Greece, where CS was not associated with AD by 36 months.^13^ Likewise, no associations were observed between CS and AD by 12 months in a German population-based prospective study of 2251 infants,^16^ nor in a Danish population-based prospective study of 3577 infants.^17^ Also, no association between CS (not defined as elective or acute) and AD by 6 years was seen in a population-based prospective study of 371 Swiss children.^18^ Finally, no certain association between CS overall and AD by age 4 was seen in a large register-based study from California.^15^ These conflicting results may be due to differences in study design, population heterogeneity, and diagnostic approach. Our study as well as a California study^15^ have a longer follow-up time than the other abovementioned studies. The children in the PreventADALL, Danish,^17^ and German^16^ studies were examined at each follow-up, while the Greek study^13^ used questionnaires for screening for AD, and the California study used diagnosis codes from electronic patient journals, potentially resulting in lower sensitivity and specificity in diagnosing AD. A long follow-up time, large study population, and thorough clinical examinations using validated diagnostic criteria for diagnosing AD may explain our detection of a positive association between CS and AD in contrast to previous studies.

Our 4 birth-mode model showed increased odds of early onset AD in children born by CS after onset of labor, which, to our knowledge, is a novel finding. The same model showed no association between any birth mode and AD by 3 years of age. Four previous studies have examined the impact of CS before or after onset of labor on AD and atopic disease overall, with results similar to ours. A register-based retrospective cohort study from the United States^19^ found no significant association between AD in the ages 3 to 10 years and uncontaminated CS (defined as repeat CS with no premature rupture of membranes) compared to contaminated CS or vaginal birth. Two small Korean studies^28^^,^^29^ of 175 and 187 children compared CS after onset of labor and elective CS to vaginal birth; they found no increased prevalence of allergic disease. A large register-based retrospective California study^15^ showed a barely significantly increased risk of AD by 4 years of age in children born by CS due to failure to progress, but it showed a modest increased risk of early onset AD (onset at 6 months) in children born by CS overall. These studies, along with ours, contradict the hypothesis that the risk for AD overall after CS is highest in children without exposure to their mother’s vaginal microbiota. The association between CS after onset of labor; the early onset AD found in our cohort could possibly be related to higher maternal stress^30^ or other maternal risk factors for CS, including pregnancy complications,^31^ with or without placental cellular stress and dysfunction.^32^^,^^33^ It is possible that early onset AD and late onset AD have different pathomechanisms, and that CS after onset of labor only increases the odds of early onset AD. However, it is outside our scope here to further explore this, so future research is needed to explain this association. The lack of association between AD by 3 years and CS before or after onset of labor in our 4 birth-mode model might also be explained by a reduction of power after we subdivided the study population into 4 delivery categories.

We did not find associations between any birth mode and persistent AD; to our knowledge, this has not been previously described. Although birth mode might influence the risk of developing AD, it does not seem to worsen the prognosis for already developed AD in early childhood, which might be reassuring for parents.

It is well established that birth mode affects formation of the infant’s microbiota,^34^, ^35^, ^36^, ^37^ so it could possibly affect the infant’s susceptibility to allergic diseases.^38^ Previous studies report that individuals with AD have different gut and skin microbial compositions compared to individuals without AD,^39^, ^40^, ^41^ although no studies have described a certain causality of this relationship. Our results showing no increase in odds for AD by 36 months, early onset AD, or persistent AD when born by CS before onset of labor suggest that lack of exposure to maternal vaginal microbiota during birth may not be important AD’s development.

Our results suggest that CS is associated with AD by 3 years, regardless of the presence of loss-of-function mutations in *FLG* and AD heredity—to our knowledge a novel finding. In contrast, the association observed between loss-of-function mutations in *FLG* and AD is well known.^9^^,^^25^ In our study, the increased odds for AD when born by CS were significant regardless of *FLG* mutation, and the increased odds were not mediated by *FLG* mutations or by heredity for AD, suggesting that CS might increase odds of AD regardless of hereditary predisposition. Lack of significant association between CS and AD by 3 years of age for infants with *FLG* loss-of-function mutations is probably due to lack of statistical power, with a small number of children in this group (n = 18). The finding that neither heredity for AD nor *FLG* mutations mediates the effect of CS on AD strengthens the generalizability of our results.

The large prospective cohort from the general population is a major strength of this study. The children attended multiple investigations during the first 3 years of life, with thorough clinical skin examinations performed by trained health care personnel, and their mothers frequently answered electronic questionnaires. We diagnosed AD using validated UK Working Party^26^ and the Hanifin and Rajka criteria,^27^ thus strengthening both the sensitivity and specificity for a correct AD diagnosis.

Some limitations need to be addressed. Although our study population is large, some subgroups included few children, limiting statistical power. Inclusion of mainly Nordic participants with a larger-than-normal prevalence of atopic diseases may reduce generalizability. The infants were born at different hospitals, potentially with different CS routines, such as use and type of pre- and perioperative antibiotics. Antibiotics are used routinely at all CS in the participating Østfold and Swedish delivery departments included in our study. In Oslo, antibiotics are routinely provided with any acute CS and at elective CS after onset of contractions and/or membrane rupture. In our study, the group of women undergoing CS in Oslo before onset of labor were therefore likely not provided antibiotics (n = 84) (results not shown). Not adjusting for receipt of antibiotics may represent another limitation. Persistent AD is challenging to define and study as a result of AD’s relapsing nature. Perhaps a different definition of persistent AD or follow-up longer than 3 years would lead to a different result. The diagnostic criteria for AD lack sensitivity to diagnose AD at 3 months, which makes early onset of AD difficult to study.

### Conclusion

CS was associated with higher odds of AD by 3 years of age regardless of infant loss-of-function *FLG* mutations or heredity for AD. To our knowledge, ours is the first prospective birth cohort study showing a positive association between CS and AD, with onset by 3 months of age, especially in children born with CS after onset of labor. We could not identify any association between birth mode and persistent AD; nor could we discover whether timing the CS before or after onset of labor alters the odds of AD by 3 years of age.

Our findings add novel information on the effect of birth mode on AD development; however, more research is needed to further explain the role of birth mode in the development of AD in early childhood.

**Key messages**

• CS was associated with increased odds for AD by 3 years of age, regardless of hereditary status for AD and the child’s *FLG* mutation status.
• CS was associated with increased odds for early onset AD
• CS was not associated with increased odds for persistent AD.

## Supplementary data

Supplementary Tables

Supplementary Figure E1
