# Supplementary material for: Birth mode is associated with development of atopic dermatitis in infancy and early childhood
Source: J Allergy Clin Immunol Glob. 2023 Mar 29;2(3):100104. doi: 10.1016/j.jacig.2023.100104 (PMC10509990; doi:10.1016/j.jacig.2023.100104)
Supplement: Supplementary Material [file mmc1.docx]

**Online repository**

Sveinung T. Hoel^1^, Johanna Wiik^2,3,4^, Karin C. L. Carlsen^1,5^, Kim M. A. Endre^1,6^, Hrefna Katrín Gudmundsdóttir^1,5^, Guttorm Haugen^1,7^, Angela Hoyer^8, 9^, Christine Monceyron Jonassen^10, 11^, Marissa LeBlanc^12^, Björn Nordlund^8, 9^, Knut Rudi^13^, Håvard O. Skjerven^1,5^, Anne Cathrine Staff^1,7^, Gunilla Hedlin^8, 9^, Cilla Söderhäll^8, 9^, Riyas Vettukattil^1,5^, Hilde Aaneland^1,5^, Eva M. Rehbinder^1,6^

**Affiliations:**

1. Institute of Clinical Medicine, Faculty of Medicine, University of Oslo, Oslo, Norway
2. Department of Gynecology and Obstetrics, Østfold Hospital Trust, Kalnes, Norway
3. Department of Obstetrics and Gynecology, Institute of Clinical Sciences, Sahlgrenska Academy, University of Gothenburg, Gothenburg, Sweden
4. Region Västra Götaland, Sahlgrenska University Hospital, Department of Obstetrics and Gynecology, Gothenburg, Sweden
5. Division of Pediatric and Adolescent Medicine, Oslo University Hospital, Oslo, Norway
6. Department of Dermatology and Vaenerology, Oslo University Hospital, Oslo, Norway
7. Division of Obstetrics and Gynaecology, Oslo University Hospital, Oslo, Norway
8. Astrid Lindgren Children’s Hospital, Karolinska University Hospital, Stockholm, Sweden
9. Department of Women’s and Children’s Health, Karolinska Institute, Stockholm, Sweden
10. Centre for Laboratory Medicine, Østfold Hospital Trust, Grålum, Norway
11. Department of Chemistry, Biotechnology and Food science, Norwegian University of Life Sciences, Ås, Norway
12. Oslo Centre for Biostatistics and Epidemiology, Oslo University Hospital, Oslo, Norway
13. Faculty of Chemistry, Biotechnology and Food Science, Norwegian University of Life Sciences, Ås, Norway

**Address of correspondence to**:

Sveinung Torsteinsen Hoel

Faculty of Medicine, Institute of Clinical Medicine, University of Oslo, Oslo, Norway.
e-mail: s.t.hoel@studmed.uio.no

Phone: +47 48426067

**Description of study design and interventions**

The PreventADALL study is an investigator-initiated 2x2 multicentre, randomized controlled clinical trial of two primary prevention interventions (skin care and early food introduction) in infancy. A total of 2697 women (2701 pregnancies) were recruited from December 2014 through October 2016. Pregnant women were recruited at the 18-week ultrasound investigation in Norway (Oslo University Hospital and Østfold Hospital Trust) and Sweden (Karolinska University Hospital, Stockholm). Women included at Karolinska University Hospital were recruited from collaborating obstetrical clinics in relation to their 18-week routine ultrasound scanning. Exclusion criteria were pregnancy with more than two fetuses, lack of sufficient Scandinavian language skill, plans to move outside reasonable travel distance within one year postpartum and severe maternal, fetal or neonatal diseases. 88.2% (n =2397) of all foetuses were included at birth with a mean (range) infant GA of 39.2 (35.6-42.9) weeks. Women carrying more than two foetuses, foetuses with severe malformations or disease and infants born prior to 35.0 weeks of GA, were excluded.

All infants were randomized at birth to one of four similar sized groups:

1. No intervention
2. Skin care (oil bath at least 5 days per week from 0.5 to 9 months of age
3. Consecutive introduction between 3 and 4 months of age, of peanut, milk, wheat and egg at least 4 days per week complementary to breastfeeding
4. Both interventions

Weekly electronic diaries (2-26 weeks of age) recorded skin care, infant feeding and symptoms of allergic disease. The infants attended follow-ups at 3, 6 and 12 months for investigation of the skin, using UK Working Party diagnostic criteria and Hanifin & Rajka diagnostic criteria to diagnose AD. The primary outcome of AD was defined as meeting the diagnostic criteria of at least one of these tools at any of the clinical visits up to 12 months of age.

Atopic dermatitis was observed in 48 (8%) of 596 infants in the no intervention group, 64 (11%) of 575 in the skin intervention group, 58 (9%) of 642 in the food intervention group and 31 (5%) of 583 in the combined intervention group (1). The primary hypotheses that either skin intervention or food intervention reduced atopic dermatitis were not confirmed (1).

Due to the results from this previous PreventADALL-study, showing no difference between the intervention groups we have not adjusted for intervention and considered the cohort as observational in this study. Sensitivity analysis adjusting for the interventions groups have been performed, showing similar results to the analyses not adjusting for interventions group (results not shown).

**Complete case analysis description:**

In the early onset analysis, there were eight missing cases before a best-case imputation was made. None of the eight attended the three-month follow-up, and all eight had an AD diagnosis. These were imputed as AD with no early onset, assuming active eczema would be motivational for follow-up attendance. In the persistent AD analysis, there were 81 missing before a best-case imputation was done. Among the 81 missing, none attended follow-up at 36 months, and all had previously diagnosed AD. Among these 81 missing, 55 had eczema by 12 months, but it is uncertain if they had eczema at 36 months or not. This group of missing participants was therefore imputed as non-persistent AD, assuming active eczema would be motivational for follow-up attendance. Results from the complete case analysis were in line with the results from the best-case imputation (results not shown).

**Region of origin of the study population:**

In our study we defined infants with *FLG-mutations (“mutation yes”)* as being carriers of any of the following mutations: R501X, 2282del4 and R2447X of the *FLG*-gene, the most common loss-of-function mutations in the European population. Most of the participating parents were of European descent as shown in Table 1. This might support that most cases of *FLG*-mutations were detected.

**References:**

1. Skjerven HO, Rehbinder EM, Vettukattil R, LeBlanc M, Granum B, Haugen G, et al. Skin emollient and early complementary feeding to prevent infant atopic dermatitis (PreventADALL): a factorial, multicentre, cluster-randomised trial. Lancet. 2020;395(10228):951-61.

**Repository legends:**

Figure E1: Direct acyclic graph for covariate analysis. The infant’s sex is associated with the risk of premature birth (E1) and also AD in prepubertal children according to a Dutch study (E2), where boys had increased risk of both caesarean section and AD in childhood. High GA increases the risk of AD (E3), and GA influences birth mode, which makes gestational age a confounding factor. Nulliparity increases the risk of AD (E3) and previous deliveries by a specific birth mode alters the chosen next birth mode, which is why having a previous delivery or not was included as a confounding factor in the adjusted logistic regression analysis. Sex (boy/girl), GA at birth (days) and parity (no previous delivery/previous delivery) was therefore adjusted for in the logistic regression model. Parental atopy, e.g., asthma is a risk factor both for atopic dermatitis in the child, but also for birth mode as asthma is strongly linked to preeclampsia, placental abruption and placenta previae and therefore also CS (E4).

**Repository references:**

E1. Yu T, Chen TS, Liang FW, Kuo PL. Does sex matter? Association of fetal sex and parental age with pregnancy outcomes in Taiwan: a cohort study. BMC Pregnancy Childbirth. 2020;20(1):348.

E2. Dirven-Meijer PC, Glazenburg EJ, Mulder PG, Oranje AP. Prevalence of atopic dermatitis in children younger than 4 years in a demarcated area in central Netherlands: the West Veluwe Study Group. Br J Dermatol. 2008;158(4):846-7.

E3. Olesen AB, Ellingsen AR, Olesen H, Juul S, Thestrup-Pedersen K. Atopic dermatitis and birth factors: historical follow up by record linkage. BMJ. 1997;314(7086):1003-8.

E4. Bonham CA, Patterson KC, Strek ME. Asthma Outcomes and Management During Pregnancy. Chest. 2018;153(2):515-27.
