# Supplementary material for: Birth mode is associated with development of atopic dermatitis in infancy and early childhood
Source: J Allergy Clin Immunol Glob. 2023 Mar 29;2(3):100104. doi: 10.1016/j.jacig.2023.100104 (PMC10509990; doi:10.1016/j.jacig.2023.100104)
Supplement: Supplementary Tables [file mmc2.docx]

Table E1: Interaction analysis between FLG-mutations and caesarean section (CS) on atopic dermatitis by 36 months. Adjusted model for sex, gestational age and parity. The analysis was conducted by including an interaction term of *FLG*-mutation by CS in a logistic regression analysis. The table shows the adjusted odds ratio (aOR) and odds ratio (OR) for the interaction effect of *FLG*-mutation by CS on AD by 36 months, which reads p > 0.05 (p=0.83 and p=0.90) indicating no evidence of an interaction effect between FLG-mutation and birth mode.

Table E2: Interaction analysis between atopic dermatitis (AD)-heredity and caesarean section (CS) on AD by 36 months. Adjusted model for sex, gestational age and parity. The analysis was conducted by including an interaction term of AD-heredity by CS in a logistic regression analysis. The table shows the adjusted odds ratio (aOR) and odds ratio (OR) for the interaction effect of AD-heredity by CS on AD by 36 months, which reads p > 0.05 (p=0.47 and p=0.50) indicating no evidence of an interaction effect between AD-heredity and birth mode.

Table E3: Stratified logistic regression model for all outcomes for atopic dermatitis (AD)-heredity group and no AD-heredity group adjusted for sex, gestational age and parity.

AD= Atopic dermatitis CS= Caesarean section

1= AD with no early onset (no eczema at 3 months, but AD by 36 months)

2= Early onset AD (eczema at 3 months and AD by 36 months)

3= Non-persistent AD (no observed eczema by 12 months of age or at 36 months investigation, and AD diagnosed by 36 months)

4= Persistent AD (eczema both by 12 months and at 36 months, and AD diagnosis by 36 months)

Table E4: Logistic regression model comparing vaginal birth (reference) against caesarean section (CS) on atopic dermatitis (AD) outcomes. Adjusted for AD-heredity, sex, gestational age and parity.

AD= Atopic dermatitis CS= Caesarean section

1= AD with no early onset (no eczema at 3 months, but AD by 36 months)

2= Early onset AD (eczema at 3 months and AD by 36 months)

3= Non-persistent AD (no observed eczema by 12 months of age or at 36 months investigation, and AD diagnosed by 36 months)

4= Persistent AD (eczema both by 12 months and at 36 months, and AD diagnosis by 36 months)

Table E5: Crosstabulation for all outcomes by birth mode (vaginal birth and caesarean section (CS)) for atopic dermatitis (AD)-heredity group and no AD-heredity group.

a= Numbers and (percentages) b= Chi square significance AD= Atopic dermatitis CS= Caesarean section

1= AD with no early onset (no eczema at 3 months, but AD by 36 months)

2= Early onset AD (eczema at 3 months and AD by 36 months)

3= Non-persistent AD (no observed eczema by 12 months of age or at 36 months investigation, and AD diagnosed by 36 months)

4= Persistent AD (eczema both by 12 months and at 36 months, and AD diagnosis by 36 months)

Table E6: Crosstabulation *FLG*-mutations by birth mode (vaginal birth and caesarean section (CS).

a= Numbers and (percentages) b= Chi square significance *FLG*= Filaggrin

Table E7: Crosstabulations *FLG-*mutations and birth mode (vaginal birth and caesarean section (CS)) by atopic dermatitis (AD)-outcomes.

a= Numbers and (percentages) b= Chi square significance

AD= Atopic dermatitis CS= Caesarean section *FLG*= Filaggrin

1= AD with no early onset (no eczema at 3 months, but AD by 36 months)

2= Early onset AD (eczema at 3 months and AD by 36 months)

3= Non-persistent AD (no observed eczema by 12 months of age or at 36 months investigation, and AD diagnosed by 36 months)

4= Persistent AD (eczema both by 12 months and at 36 months, and AD diagnosis by 36 months)

Table E8: Baseline characteristics of the study population included in this substudy from the PreventADALL mother-child cohort compared to the participants that were not included in this study population.

AD= Atopic dermatitis CS= Caesarean section SD= Standard deviation BMI= Body mass index

1= From the questionnaire at enrolment

2= From the questionnaire at 36 weeks

a= Numbers (percentages)

b=Chi square-significance level
