# Supplementary figures and images for: Birth mode is associated with development of atopic dermatitis in infancy and early childhood
Source: J Allergy Clin Immunol Glob. 2023 Mar 29;2(3):100104. doi: 10.1016/j.jacig.2023.100104 (PMC10509990; doi:10.1016/j.jacig.2023.100104)

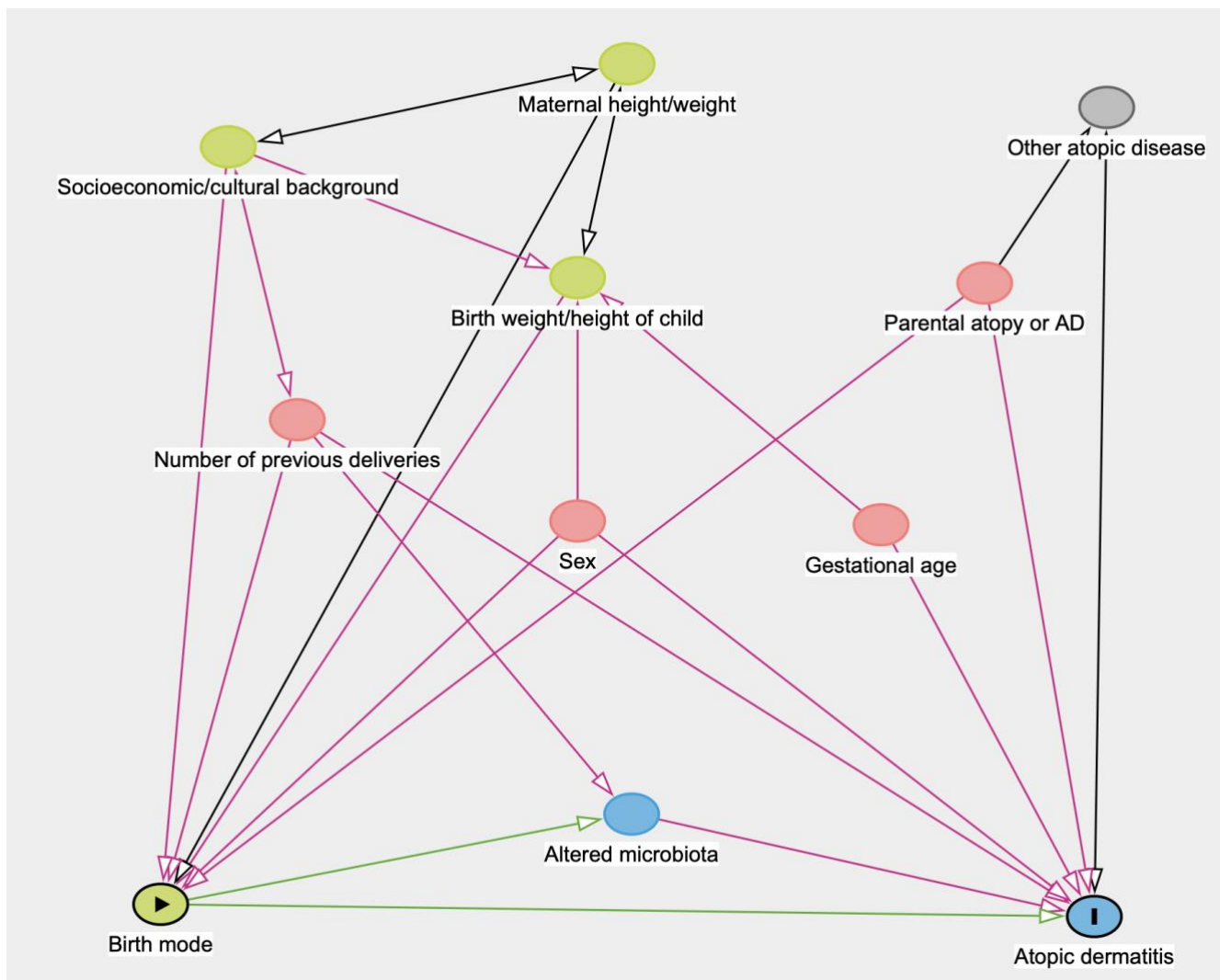

Supplement: Supplementary Figure E1 [file mmc3.pdf]
